# Data on students’ mathematical reasoning test scores: A quasi-experiment

**DOI:** 10.1016/j.dib.2020.105546

**Published:** 2020-04-17

**Authors:** Angel Mukuka, Vedaste Mutarutinya, Sudi Balimuttajjo

**Affiliations:** a African Centre of Excellence for Innovative Teaching and Learning Mathematics and Science, University of Rwanda-College of Education; b University of Rwanda-College of Education, Department of Mathematics, Science and Physical Education; c Department of Educational Foundations and Psychology, Mbarara University of Science and Technology, Uganda

**Keywords:** Conjecturing, Justifying, Mathematical reasoning, Mathematizing, Quasi-experiment

## Abstract

The data presented here were collected from 301 grade 11 students selected from six public secondary schools within one district. These students participated in a quasi-experimental study whose aim was to investigate the effects of the Student-Teams Achievement Division (STAD) on students’ mathematical reasoning. Six intact classes from the participating schools were selected using a cluster random sampling method, three of which were randomly assigned to the control group while the other three were randomly assigned to the experimental group. Respondents’ demographic information (identity, gender, school type, and age) are presented alongside their raw scores on pretest and posttest measures. The mathematical reasoning test (MRT) items presented to students aimed at assessing their conjecturing, justifying and mathematizing abilities. Student scores from each of these three mathematical reasoning dimensions were aggregated to form a total score for both the pretest and posttest measures. These data can provide some insights into the extent to which different learning conditions can support the development of reasoning among the learners of school mathematics. The MRT items presented in the supplementary data files can also act as a basis for the formulation of new tasks aimed at assessing students’ mathematical reasoning skills.

Specifications table

**Table:** 

Subject	Mathematics Education
Specific subject area	Mathematical Reasoning
Type of data	Table Graph Figure
How data were acquired	Data were acquired via a Mathematical Reasoning Test (MRT) on Quadratic Equations and Quadratic Functions. The test was administered to 301 grade 11 students before and after the intervention
Data format	Raw
Parameters for data collection	Six grade 11 intact classes were randomly selected from six secondary schools clustered into low, moderate and high average performance, and then randomly assigned to the control and experimental groups. The control group was taught using the conventional methods of teaching (mainly expository teaching) whereas the experimental group was taught using the Student-Teams Achievement Division (STAD) model of cooperative learning.
Description of data collection	After the random assignment of six intact classes to experimental and control groups, a pretest was administered followed by a series of lessons on quadratic equations and quadratic functions under the learning conditions prescribed above. These lessons lasted for six weeks after which a posttest was administered to the same students. Student test scores were recorded for both the pretest and posttest. Students’ conjecturing, justifying and mathematizing ability levels for the posttest were also recorded as indicated in the dataset (MR Dataset. sav).
Data source location	Town/Region: Ndola District, Copperbelt Province Country: Zambia
Data accessibility	The dataset named “MR Dataset. sav “ is openly available on Mendeley Data Repository at http://dx.doi.org/10.17632/3472zggczv.1

## Value of the data

• These data can provide some insights into appropriate instructional and assessment avenues for enhancing students’ mathematical reasoning skills.
• The data are useful and beneficial to mathematics education researchers, and teachers of school mathematics.
• The mathematical reasoning test items can serve as a basis for the development of new mathematical tasks aimed at assessing students’ abilities in conjecturing and justifying various algebraic statements/arguments as well as assessing students’ abilities in linking classroom mathematics to real-world experiences and vice versa.
• Besides being a basis for new theory development, these data can be used for replication studies in mathematics education research and practice

## 1 Data description

The quasi-experimental data described here were collected via a mathematical reasoning test (MRT) that comprised a total of 16 test items organized under 7 questions. The 16 MRT items were allocated to three mathematical reasoning dimensions (conjecturing, justifying and mathematizing) after a critical review by experts. A detailed description of these dimensions, the validation process, and related documents are provided in the supplementary materials. Table 1 shows the specific test items allocated to each of the three dimensions, while the actual items are presented in the supplementary file named MRT.pdf. Formulation of the final version of the mathematical reasoning test (MRT.pdf) was based on the expert comments and suggestions regarding the initial test items of the MRT_before validation.pdf that was submitted to them for validation.

**Table 1:** Finalized Mathematical Reasoning (MR) dimensions and allocated MRT items

MR Dimension	Question number															
	*1(a)*	*(b)*	*2*	*3(a)*	*(b)*	*4(a)*	*(b)*	*5(a)*	*(b)*	*6(a)*	*(b)*	*7(a)*	*(b)*	*(c )*	*(d)*	*(e )*
Conjecturing			✓	✓		✓				✓				✓		
Justifying	✓		✓	✓	✓		✓			✓			✓	✓		
Mathematising		✓						✓	✓	✓	✓	✓			✓	✓

The MR Dataset.sav (available on http://dx.doi.org/10.17632/3472zggczv.1), includes participant scores for each of the three dimensions whose totals were aggregated to form the total MRT score for each of the measures (pretest and posttest). Demographic variables such as a respondent's identity, gender, school type, and age have equally been specified in the dataset. It should be noted that the data stored in the last 4 columns of the MR Dataset.sav are not of interest to this paper.

After administration and marking of the posttest, students’ ability levels for each of the three dimensions (conjecturing, justifying and mathematizing) were determined as “adequate or inadequate” as prescribed in the variable view of the MR Dataset.sav file. Students who scored less than 50% for each of the three dimensions were classified under the ‘inadequate reasoning’ category while those who scored 50% or more were classified under the ‘adequate reasoning’ category. This categorization is based on the standard pass rate criteria set by the Zambian tertiary institutions. Scores falling below 50% at school certificate level are classified as not being adequate enough to warrant a college or university place for all individuals seeking to enroll for various study programs.

## 2 Experimental design, materials, and methods

### 2.1 Formulation and development of the MRT items

The MRT items were formulated in line with previous studies [1,2] on mathematical reasoning for school mathematics as well as the aims and objectives of the Zambian school mathematics curriculum [3]. The mathematical tasks presented by Norqvist, Jonsson, and Lithner [4] also demonstrate that assessment of mathematical reasoning skills should not be restricted to the logical analysis of mathematical conjectures/arguments but also to understand students’ ability to apply classroom mathematics to real-life situations and vice versa. Before administration to the intended respondents, the MRT items were validated by 13 experts who were purposively selected for the critical review of all the test items.

A detailed description of the validation procedure is given in the guidelines that were sent to the validators. These guidelines (MRT validation guidelines.pdf) together with a letter to the expert (Letter to the expert.pdf), initial test items (MRT_before validation.pdf), and the validation sheet (MRT validation sheet) are available in the supplementary data files provided. The validators were chosen and contacted via email or face-to-face where possible. They were chosen because of their vast research experience, teaching experience and their knowledge of the Zambian mathematics curriculum for secondary schools or other contexts with similar education systems. Based on comments and suggestions from experts, the test items were revised and sent back to them for a final review. Thereafter, a final version (MRT.pdf) was compiled and then used as a data collection instrument.

### 2.2 Ethical considerations

Before data were collected from the intended respondents, permissions from relevant authorities (Data collection permit.pdf) were sought and granted. All the participants provided written consent (Consent form_students.pdf) and the study had received ethical approval from the Research and Innovations Directorate of the College of Education, University of Rwanda.

### 2.3 Sampling and data collection procedures

The data described in the previous section (data description) were collected from 301 grade 11 students of ages 14 to 20 (*M* = 16.29, *SD* = 1.00). Of this number, 97 (32.2%) students were male while 204 students were female. The reason for having more female students than male students could be attributed to the fact that one of the randomly selected schools was a girls’ school. It was further noted that even schools that comprised both male and female students had more female than male students. A cluster random sampling method was used to select the six classes from each of the six participating public schools within the district. Based on the information obtained from the district education board secretary's office, the 20 public secondary schools were clustered into low, moderate and high academic performance using the national examinations mean performance for each school. Two schools were then randomly selected from each cluster and randomly assigned to the control and experimental groups. Thereafter, one grade 11 class was randomly selected from each of the participating schools, and all the students from each selected class were included in the sample. This means that each of the two groups (control and experimental) had representation from each of the three clusters. All the respondents participated in a pretest to establish the equivalence of the two groups (control and experimental) in terms of their mathematical reasoning abilities before the intervention. In line with a constructivist view of learning a pretest was administered to understand students’ existing knowledge on quadratic equations and quadratic functions.

Based on the sampling criteria prescribed above, 97 (32.2%) of these participants came from low performing schools while 123 (40.9%) came from moderate performing schools and 81 (26.9%) came from high performing schools. Students’ mathematical reasoning abilities in relation to the type of school average performance on MRT items 3 and 4 of the MRT.pdf have been reported in descriptive survey research that was carried out prior to the intervention [5]. After a random allocation of six schools to two groups (control and experimental), it was found that 150 (49.8%) students were assigned to the control group while 151(51.2%) were allocated to the experimental group.

### 2.4 Experimental conditions and procedures

After the administration of pretest to both groups, the experimental group was taught using the Student-Teams Achievement Division (STAD) as prescribed by [6, 7], while the control group was taught using conventional methods of teaching. The commonly observed instructional approach in the control group was mainly expository, characterized by “chalk and talk” and what Slavin [8] referred to as a “standard hear lecture–do problems–get feedback order of affairs”. After learning quadratic equations and quadratic functions for six weeks under these conditions, students in both groups took the MRT test (posttest). Students’ responses to the test items were evaluated and the scores (pretest and posttest) were recorded as indicated in the MR Dataset.sav available on http://dx.doi.org/10.17632/3472zggczv.1.

## Appendix Supplementary materials

Image, application 1

Image, application 2
