# Supplementary material for: Data on students’ mathematical reasoning test scores: A quasi-experiment
Source: Data Brief. 2020 Apr 17;30:105546. doi: 10.1016/j.dib.2020.105546 (PMC7176821; doi:10.1016/j.dib.2020.105546)
Supplement: Supplementary file 1 [file mmc1.zip › Supplimentary files/Consent form_students.pdf]

CONSENT FORM FOR STUDENTS

RESEARCH TITLE: ***Effects of Cooperative Learning on Students' Mathematical Reasoning and Self-efficacy in Selected Secondary Schools, Ndola District, Zambia.***

RESEARCHER'S NAMES: ***Angel MUKUKA***

I have been given information about the purpose of the study and discussed the research project with ***Angel MUKUKA*** who is conducting this research as part of a ***Doctor of Philosophy (Ph.D.) in Mathematics Education*** being undertaken at the ***African Centre of Excellence for Innovative Teaching and Learning Mathematics and Science (ACEITLMS)***, University of Rwanda, College of Education.

I understand that my participation in this research is voluntary, I am free to refuse to participate and I am free to withdraw from the research at any time. If I have any enquiries, concerns or complaints regarding the way the research is or has been conducted, I can contact the Research and Ethics Committee of the University of Rwanda, College of Education or ACEITLMS on email *aceitlms@ur.ac.rw* .

By signing below I am indicating my consent to (please tick):

- Voluntarily attend mathematics lessons even outside my class time table but within my school premises during the day, where necessary.
- Participate in mathematical reasoning assessments during data collection
- Allow copies of my test scripts to be used in the study
- Cooperate with my teacher and the researcher during mathematics lessons and test sessions
- My own identity, my teacher's identity and that of my school will remain confidential.
- Withdraw from the study at any point should I feel uneasy about the study.
- Decline to answer any question in the questionnaire, test or during interviews.
- My voice being recorded (where necessary) when I am interviewed.

I understand that the data collected from my participation will be used for ***academic purposes only*** *(Ph.D. thesis, conference proceedings and journal publications)* and I consent for it to be used in that manner.

Signed ***Date***

_______________________________________ _______/_______/________

NAME (IN CAPITAL LETTERS)

SCHOOL/CLASS____
