# Supplementary material for: Data on students’ mathematical reasoning test scores: A quasi-experiment
Source: Data Brief. 2020 Apr 17;30:105546. doi: 10.1016/j.dib.2020.105546 (PMC7176821; doi:10.1016/j.dib.2020.105546)
Supplement: Supplementary file 1 [file mmc1.zip › Supplimentary files/Data collection permit.pdf]

UNIVERSITY OF RWANDA

COLLEGE OF EDUCATION

RESEARCH AND INNOVATION UNIT

23rd November, 2018
Ref: 01/P-CE/.458./EN/gi/2018

The Permanent Secretary,
Ministry of General Education
P.O.Box: 50093
Lusaka-Zambia

REPUBLIC OF ZAMBIA MINISTRY OF GENERAL EDUCATION
18 MAR 2019
DISTRICT EDUCATION BOARD SECRETARY NDOLA DISTRICT EDUCATION BOARD P.O.BOX 71979, NDOLA

Dear Sir/Madam,

Re: **Recommendation for Mr. Angel Mukuka to conduct a research study in Zambia**

Mr. Mukuka, a citizen of Zambia, is currently a doctoral student of the African Centre of Excellence for Innovative Teaching and Learning Mathematics and Sciences (ACEITLMS) at the University of Rwanda-College of Education. As a PhD by research student, he is conducting a research entitled: "**Effects of Cooperative learning on students' Mathematical Reasoning and self-efficacy in selected secondary schools, Ndola District, Zambia**". His research will involve grade 11 students and their teachers of mathematics from selected schools located in Ndola district of zambia.

Mr Mukuka's research project passed through an internal collegial ethical process. Thus, the University of Rwanda-College of Education: Directorate of research and Innovation confirms that his research adheres to ethical standards and principles. Therefore, we kindly request you to consider his application for authorization to conduct research in Zambia, and provide him with necessary support he may require to successfully undertake his study.

Your permission for him to conduct the study will be highly appreciated.

Yours sincerely,

UNIVERSITY OF RWANDA - COLLEGE OF EDUCATION - CE

Assoc. Prof. Eugene Ndabaga
Director of Research and Innovation
University of Rwanda- College of Education
E-mail: ndabagav@yahoo.ie
Mobile: +250788308862

Cc:

- The Principal,UR-CE
- Director, ACEITLMS, UR-CE

All communications should be addressed to:
The Permanent Secretary, Ministry of General Education
Not to any individual by name

Telephone: 250855/251315/251283
251293/211318/251291
251003/251319

In reply please quote

TS: 803424

REPUBLIC OF ZAMBIA

MINISTRY OF GENERAL EDUCATION

P. O. BOX 50093
LUSAKA

31st January, 2019

Mr. Angel Mukuka
African Centre of Excellence for Innovative & Learning Mathematics and Science
College of Education,
University of Rwanda

RE: REQUEST FOR AUTHORITY TO COLLECT DATA FROM SELECTED SECONDARY SCHOOLS IN NDOLA DISTRICT.

Refer to your request dated 17th December 2018.

I am pleased to inform you that your request to collect data from some selected Secondary schools in Ndola District has been granted.

Upon receipt of this letter, you proceed to make arrangements with the Provincial Education Officer Copperbelt.

Kindly note that your research should not in any way interfere with learning programs in the schools.

Dr. Jobbicks Kalumba
Permanent Secretary
Ministry of General Education

/cjm..

All communications should be addressed to the Provincial Education Officer

Telephone: 0212 615416
0212 615625

In reply please quote:

No.:........................

REPUBLIC OF ZAMBIA

MINISTRY OF GENERAL EDUCATION

OFFICE OF THE PROVINCIAL EDUCATION OFFICER
P.O. Box 71552
Ndolla - Zambia

14th March 2018

The District Education Board Secretary
NDOLA DISTRICT

RE: INTRODUCTORY LETTER: ANGEL MUKUKA

This serves to introduce the above mentioned student currently pursuing Doctoral studies in Mathematics Education at African Centre of Excellence for innovative Teaching and Learning Mathematics and Science, University of Rwanda, College of Education.

I write to inform you that authority has been granted for him to conduct a study Ndola District Schools.

Kindly ensure that his study does not interfere with teaching and learning.

REPUBLIC OF ZAMBIA
MINISTRY OF GENERAL EDUCATION
PRINCIPAL EDUCATION STANDARDS OFFICER
14 MAR 2019
P.O. BOX 71552,
[illegible]
COPPERBELT PROVINCE

Ngoma Felix Z.
for/ Provincial Education Officer
COPPERBELT PROVINCE

/nnt
