# Supplementary material for: Data on students’ mathematical reasoning test scores: A quasi-experiment
Source: Data Brief. 2020 Apr 17;30:105546. doi: 10.1016/j.dib.2020.105546 (PMC7176821; doi:10.1016/j.dib.2020.105546)
Supplement: Supplementary file 1 [file mmc1.zip › Supplimentary files/Letter to the expert.pdf]

African Centre of Excellence for Innovative Teaching & Learning Mathematics and Science (ACEITLMS), College of education, University of Rwanda.
E-mail: mukukaangel@yahoo.com
Phone: +250780788343/+260954027181

Dear respected scholar/educator,

As part of my PhD research project I am currently at the phase of carrying out a content/construct validation of a test to assess students' mathematical reasoning. The focus is on Grade 11 students' ability to make justified inferences and their conceptual understanding of quadratic equations and quadratic functions.

Your assistance as an expert in Mathematics Education at this stage of the development of such an instrument is crucial and it will sincerely be appreciated.

Attached to this letter are the following documents:

1. Mathematical Reasoning Test (Test questions on Quadratic equations & quadratic functions)
2. Guidelines for instrument validation and
3. Excel validation sheet

Kindly, read the guidelines before you provide the ratings in the attached excel sheet. Also refer to the specific test items as you make your own judgement about the ratings. For any clarification, do not hasitate to contact the principal investigator or any of the advisors listed below:
Thanking you in anticipation

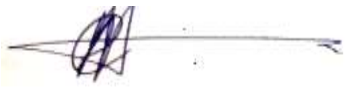

Angel MUKUKA
<u>Ph.D. Scholar.</u>

SUPERVISORS' DETAILS

Dr. Vedaste MUTARUTINYA
Senior Lecturer of Mathematics, University of Rwanda, College of Education, +250788540984 or email vedastemuve@yahoo.fr

Dr. Sudi BALIMUTTAJJO
Senior Lecturer of Mathematics Education, Mbarara University of Science & Technology, Uganda. +256752159426 or Email sudib@must.ug
