# Supplementary material for: Data on students’ mathematical reasoning test scores: A quasi-experiment
Source: Data Brief. 2020 Apr 17;30:105546. doi: 10.1016/j.dib.2020.105546 (PMC7176821; doi:10.1016/j.dib.2020.105546)
Supplement: Supplementary file 1 [file mmc1.zip › Supplimentary files/MRT validation guidelines.pdf]

GUIDELINES FOR MRT VALIDATION PROCEDURE

A. DEFINITIONS OF DIMENSIONS

Reasoning is part of a much wider set of skills that are required to help us develop mathematically and allow us to think critically. Therefore, reasoning skills should be cherished and be an integral part of learning mathematics for all secondary school students. The ability to communicate lies at the heart of reasoning and this is something that we as teachers, need to really encourage. In this study students are considered to reason mathematically when they explain their thinking, when they deduce and justify strategies used and conclusions reached, when they adapt the known to unknown, when they transfer learning from one context to another, when they explain why something is true or false and when they compare and contrast related mathematical concepts.

Besides being highly deductive in nature, the mathematical reasoning being implied in this research also involves students' ability to relate classroom mathematics to real world experiences or vice versa. In addition, inductive reasoning is considered central to the formulation of algebraic conjectures, which would eventually be justified through deductive reasoning. In view of the above, students' mathematical reasoning is assessed using the following mathematical proficiencies referred herein as *'dimensions'* of mathematical reasoning:

Conjecturing

Conjecturing involves reasoning about mathematical relationships to develop statements that can be proven right or wrong based on established mathematical facts. This dimension includes questions requiring students to demonstrate understanding of facts and ideas as well as examining and breaking information into parts by identifying patterns, motives or cause. By so doing, students would be able to make intelligent guesses that would eventually be justified through a logical chain of steps in arriving at a conclusion.

Justifying and validating mathematical statements and arguments

In this dimension, we included questions that require students to make inferences and find evidence to support generalizations. They are expected to present and defend opinions by making judgments about information, validity of ideas, or quality of work based on a set of criteria.

Applying and mathematising

This dimension involves questions that require students to solve problems by applying acquired knowledge, facts, techniques and rules. It encompasses the process of using a rational and systematic series of steps based on sound mathematical procedures and given statements to arrive at the answer. Questions requiring students to make connections of mathematical concepts to real world experiences or the other way round were paramount in this dimension.

B. RATINGS CRITERIA

According to the following indicators, ratings and description, please rate each of the test items using the attached **excel validation sheet**.

Indicator	Rating	Description
Sufficiency The items belong to the same dimension and are adequate to measure a particular dimension.	1	Items are not enough to measure the dimension
	2	Items measure some aspect of the dimension but do not correspond to the entire dimension
	3	Some items must be increased in order to evaluate the dimension completely
	4	The items are sufficient
Clarity The item is well articulated and can be understood easily by grade 11 students in the Zambian mathematics curriculum for secondary schools.	1	The item is not clear
	2	The item requires a big deal of modifications in the use of the words according to their meaning.
	3	The item requires a specific modification in the use of words according to their meaning
	4	The item is clear, has appropriate semantics and syntax
Coherence The item is logically related to the dimension it is intended to measure	1	The item has no logical relation to the dimension
	2	The item has a tangential relationship with the dimension
	3	The item is reasonably related to the dimension
	4	The item is completely related to the dimension
Relevance The item is very essential and important in measuring students' reasoning ability and conceptual understanding	1	The item can be eliminated without affecting the measurement of the dimension
	2	The item has some relevance, but another item may be including what this one measures.
	3	The item is relatively important
	4	The item is very relevant and should be included

** Note: Only scores (1, 2, 3 or 4) should be entered on the item excel validation sheet attached.*

C. SPECIFIC OUTCOMES FOR "QUADRATIC EQUATIONS & QUADRATIC FUNCTIONS" IN THE ZAMBIAN O' LEVEL SYLLABUS

TOPIC	SUBTOPIC	SPECIFIC OUTCOME	SKILLS	VALUES
**Quadratic Equations**	Introduction to Quadratic equations<br><br>Solutions of quadratic equations	(i). Explain the meaning of the quadratic equation<br>(ii). Solve quadratic equations by graphical method<br>(iii). Solve quadratic equations using factorisation method<br>(iv). Solve quadratic equations using completing of square method<br>(v). Solve quadratic equations using quadratic formula method<br>(vi). Apply quadratic equations to solve real life problems	***Identification*** of method of quadratic equations<br><br>***Computation*** of quadratic equations using various methods.	***Logical thinking*** in computing/solving quadratic equations.<br>***Accuracy*** in finding quadratic roots.
**Quadratic Functions**	Introduction to quadratic functions	(i). Explain the quadratic function and its graph<br>(ii). Sketch the graph of a quadratic function	***Identification*** of a quadratic function.<br>***Interpretation*** of Maximum and minimum values of a function.<br>***Drawing*** of function graphs.	***Neatness*** in sketching graphs.<br>***Logical thinking*** in determining the turning points.<br>***Accuracy*** in finding the roots.

Source: Ordinary level mathematics syllabus
