# Supplementary material for: Data on students’ mathematical reasoning test scores: A quasi-experiment
Source: Data Brief. 2020 Apr 17;30:105546. doi: 10.1016/j.dib.2020.105546 (PMC7176821; doi:10.1016/j.dib.2020.105546)
Supplement: Supplementary file 1 [file mmc1.zip › Supplimentary files/MRT.pdf]

MATHEMATICAL REASONING TEST FOR GRADE 11 STUDENTS

INSTRUCTIONS
*Answer all the questions completely using the spaces provided. You are advised to spend no more than **45** minutes to complete this paper.*

PART 1: DEMOGRAPHIC INFORMATION

Students' name: ____

Gender (Male or Female):____

Age (in years): ____

PART 2: QUESTIONS

1. Given that $x = t + 1$ and $y = t^2 + t$.
   (a) Show that $4x^2 - 3y = t^2 + 5t + 4$.
   (b) Hence, determine the value of t for which $8x^2 - 6y = 20$ given that $t > 0$

____
____
____
____
____
____
____
____
____
____
____
____
____

2. State the condition for which the roots of a quadratic equation will be non-real. Give a reason for your answer.

____
____
____
____
____
____
____
____

3. Your teacher of mathematics asked the class to solve the equation $(x + 2)(x - 3) = 14$. Three of your classmates gave the following solutions:

Student A	*Student B*	*Student C*
$(x+2)(x-3)=14$<br>$x+2=14$ *or* $x-3=14$<br>$x=14-2$ *or* $x=14+3$<br>$x=12$ *or* $x=17$	$(x+2)(x-3)=14$<br>$x^2-3x+2x-6=14$<br>$x^2-x-6=14$<br>$x^2-x-20=0$<br>$x^2-4x+5x-20=0$<br>$x(x-4)+5(x-4)=0$<br>$(x+5)(x-4)=0$<br>$x+5=0$ *or* $x-4=0$<br>$x=-5$ *or* $x=4$	$(x+2)(x-3)=14$<br>$x^2-3x+2x-6=14$<br>$x^2-5x-6=14$<br>$x^2-5x=20$<br>$x^2-5x-20=0$<br>$x=\frac{-b\pm\sqrt{b^2-4ac}}{2a}$<br>$=\frac{-5\pm\sqrt{-5^2-4(1)(-20)}}{2(1)}$<br>$\frac{-5+\sqrt{-25+80}}{2}$<br>$=\frac{-5+\sqrt{55}}{2}$ *or* $\frac{-5-\sqrt{55}}{2}$<br>$x=1.21$ *or* $x=-6.21$

(a) Determine whether each of the solutions above is *correct* or *wrong* by ticking the appropriate box in the table below. Based on your choice, point out what went wrong in the solution or justify why you think the solution is correct.

	Correct	***Wrong***	***Reason(s)***
**Student A**			
**Student B**			
**Student C**			

(b) If none of the three solutions is correct, provide your own solution and justify why you think it is the right one.

____

____

____

____

____

4. Consider the statement ***"x^2+1 can never be zero"***.

(a) If x is a real number, state whether the above statement is **true** or **false**

(b) Justify your choice in (a) above

____

____

____

____

____

5. The diagram below shows a sketch of the graph of $y = x^2 - 6x + 8$, cutting the y-axis at A and the x- axis at B and C.

(a) Find the coordinates of **A, B** and **C**

(b) Find the coordinates of the turning point of the graph.

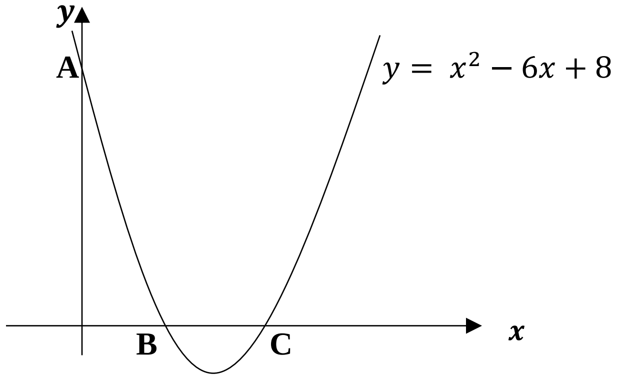

6. A boy buys x eggs at $(x - 8)kwacha$ each and $(x - 2)$ note books at $(x - 3)$ $kwacha$ each. If the total bill is 76 $kwacha$;

(a) Show that $2x^2 - 13x - 70 = 0$

(b) Hence determine the number of eggs and the number of note books that he bought

7. A farmer has $60m$ of wire fencing which he uses to make a rectangular pen for his sheep. He uses a stone wall as one side of the pen so that the wire is used for only 3 sides of the pen as shown in the diagram below:

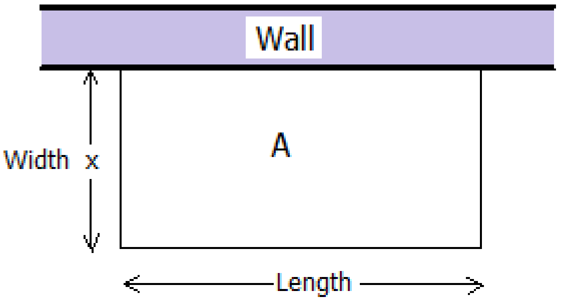

(a) If the width of the pen is x m, determine the length of the pen, in terms of x

(b) Hence, show that the area, A of the pen is given by $A = 60x - 2x^2$.

(c) State whether the function $A = 60x - 2x^2$ will have a ***maximum*** or ***minimum*** value. Give a reason for your choice.

(d) Sketch the graph of $A = 60x - 2x^2$ taking values of x from 0 to 30.

(e) What dimensions should the pen have if the farmer wants to enclose the largest possible area?

THANK YOU FOR YOUR PARTICIPATION!
