# Supplementary material for: Data on students’ mathematical reasoning test scores: A quasi-experiment
Source: Data Brief. 2020 Apr 17;30:105546. doi: 10.1016/j.dib.2020.105546 (PMC7176821; doi:10.1016/j.dib.2020.105546)
Supplement: Supplementary file 1 [file mmc1.zip › Supplimentary files/MRT_before validation.pdf]

MATHEMATICAL REASONING TEST FOR GRADE 11 STUDENTS

INSTRUCTIONS

Answer all the questions completely using the spaces provided. You are advised to spend ***45*** *minutes to complete this paper.*

PART 1: DEMOGRAPHIC INFORMATION

Students' name: ____

Name of School: ____

Gender (Male or Female): ____

Age (in years): ____

PART 2: QUESTIONS

1. Given that $x^2 + y^2 = 7$ and that $(x+y)^2 = 27$,
   (a) Show that $xy = 10$
   (b) Hence, find the numerical value of $(x-y)^2$

2. (a) The solutions of the equation $ax^2 + bx + c = 0$ are given by $x = \frac{-b \pm \sqrt{b^2-4ac}}{2a}$. State the condition for which these solutions will be non-real. Give a reason for your answer.
   (b) Consider the statement **"$\boldsymbol{x^2 + 1}$ *can never be zero*".** Prove whether this statement is true or false given that x is a real number.

3. Your teacher of mathematics asked the class to solve the equation $(x+2)(x-3) = 14$. Three of your classmates gave the following solutions:

Student A	*Student B*	*Student C*
$(x+2)(x-3)=14$<br>$x+2=14\ OR\ x-3=14$<br>$x=14-2\ \ OR\ x=14+3$<br>$x=12\ \ OR\ \ x=17$	$(x+2)(x-3)=14$<br>$x^2-3x+2x-6=14$<br>$x^2-x-6=14$<br>$x^2-x-20=0$<br>$x^2-4x+5x-20=0$<br>$x\,(x-4)+5(x-4)=0$<br>$(x+5)(x-4)=0$<br>$x+5=0\ \ OR\ \ x-4=0$<br>$x=\ -5\ \ OR\ x=4$	$(x+2)(x-3)=14$<br>$x^2-3x+2x-6=14$<br>$x^2-5x-6=14$<br>$x^2-5x=20$<br>$x^2-5x-20=0$<br>$x=\frac{-b\pm\sqrt{b^2-4ac}}{2a}$<br>$=\frac{-5\pm\sqrt{-5^2-4(1)(-20)}}{2(1)}$<br>$\frac{-5+\ \sqrt{-25+80}}{2}$<br>$=\frac{-5+\ \sqrt{55}}{2}\ OR\ \frac{-5-\sqrt{55}}{2}$<br>$x=1.21\ \ OR\ \ x=\ -6.21$

(a) Determine whether each of the solutions above is *correct* or *wrong* by ticking the appropriate box in the table below. Based on your choice, point out what went wrong in the solution or justify why you think the solution is correct.

	Correct	***Wrong***	***Reason(s)***
**Student A**			
**Student B**			
**Student C**			

(b) If none of the three solutions is correct, provide your solution and justify why you think your solution is right.

4. The diagram below shows a sketch of the graph of $y = x^2 - 6x + 8$, cutting the y-axis at A and the x- axis at B and C.

   (a) Find the coordinates of **A, B** and **C**

   (b) Find the coordinates of the turning point of the graph.

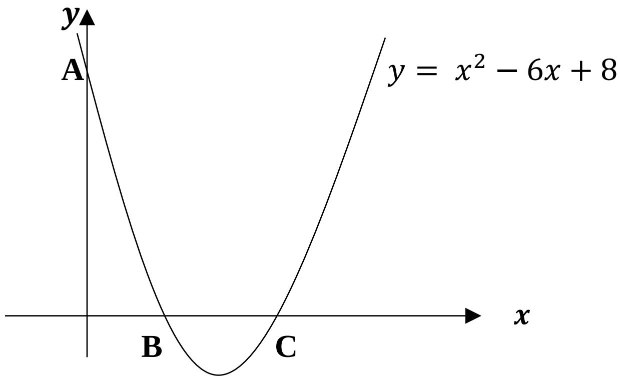

5. A boy buys x eggs at $(x - 8)kwacha$ each and $(x - 2)$ note books at $(x - 3)$ $kwacha$ each. If the total bill is 76 $kwacha$;

   (i) Show that $2x^2 - 13x - 70 = 0$

   (ii) Hence determine the number of eggs and the number of note books that he bought

6. A farmer has $60m$ of wire fencing which he uses to make a rectangular pen for his sheep. He uses a stone wall as one side of the pen so that the wire is used for only 3 sides of the pen as shown in the diagram below:

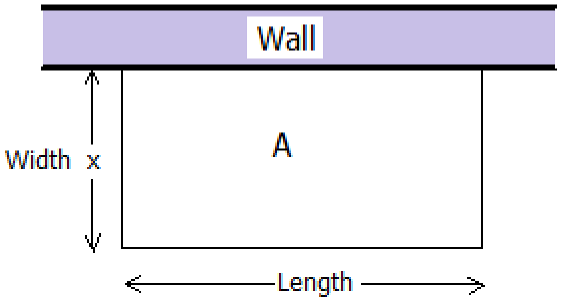

(a) If the width of the pen is x m, determine the length of the pen, in terms of x

(b) Hence, show that the area, A of the pen is given by $A = 60x - 2x^2$ (Justify every step in your solution).

(c) Sketch the graph of $A = 60x - 2x^2$ taking values of x from 0 to 30.

(d) What dimensions should the pen have if the farmer wants to enclose the largest possible area?

END OF TEST!
